# Use of Digital Technologies in Home Office Work during the COVID-19 Pandemic

**DOI:** 10.2174/17450179-v18-e2208190

**Published:** 2022-08-26

**Authors:** Lucio Lage Gonçalves, Antonio Egidio Nardi, Hugo dos Santos, Douglas Rodrigues, Anna Lucia Spear King

**Affiliations:** 1 Delete Lab. Digital Detox and Conscious Use of Technologies, Institute of Psychiatry IPUB), Federal University of Rio de Janeiro (UFRJ). Av. Venceslau Bras 71, Botafogo - Rio de Janeiro, 22290 Brazil; 2 Department of Mathematics and Statistics, Institute of Statistics, Fluminense Federal University (UFF), Rio de Janeiro, Brazil

**Keywords:** Home office, Coronavirus, COVID-19, Pandemic, Human behavior, Social distancing

## Abstract

**Background:**

Social distancing as a preventive measure to contain the spread of the COVID-19 pandemic has resulted in many people working from home, using online digital resources. Staying at home has led to the adaptation of many work activities to allow continuity of people´s jobs. It can also affect home routines and ways of working, thereby leading to changes in behavior, as the main interest of this study.

**Objective:**

The study aimed to assess the impact on human behavior of working conditions in home office format due to social distancing.

**Methods:**

Data collection was done online, using a specific computational tool (Google Forms) for this type of research, using the Home Office Work Scale (HOWS) validated and published in *Mental Health and Addiction Research* in 2021, with a total sample of 1,056 valid questionnaires. After the data collection, a database was created for statistical analysis of the results.

**Results:**

More women than men volunteered to answer the questionnaire, although the results were similar between women and men. Home office work has impacts on human behavior and results in changes in routines and adaptations in people´s personal and professional lives.

**Conclusion:**

Proportionally, more women participated, and there was low participation by young and elderly people. In general, people accepted home office work and the possibility of continuing to work in this format. Changes to routines and restrictive adaptations were necessary. The limitations reported for applying the scale did not compromise the results.

## 1 INTRODUCTION

 Social distancing due to the novel coronavirus (SARS-CoV-2) pandemic has changed people`s routines. Staying at home has transformed behaviors in the consumption of products and services. Many people have had to adapt their practices, such as communicating virtually while staying at home, involving long hours connected to digital devices.

 Social isolation tends to provoke psychological reactions such as increased levels of anxiety, stress, and irritability, the appearance of fears (based on real or subjective information), and confused thinking, negatively affecting the individual's ability to make coherent decisions [1, 2].

The impacts of social isolation on mental well-being are well known. Isolation and loneliness, among other behaviors and feelings, tend to affect individuals and those around them, and this is especially the case during the COVID-19 pandemic [3].

Abusive internet use has negative impacts, affecting work performance, academic life, family life, social relations, physical health, and psychological well-being [4].

Working at home is different from working in the normal workplace. For example, the regular workplace routine with breaks during the workday and face-to-face conversation no longer exist. Many people miss such contacts.

Digital technologies can change the way people form relationships and socialize with others, with positive and negative effects; it all depends on how such technologies are used or abused [5].

Interpersonal relations change in the home office, and such conditions can produce dynamics leading to physical and psychological harm.

Excessive use of computers, cellphones, tablets, and other devices has favored the emergence of disorders and more frequent functional limitations, identified by clinicians [6].

Loneliness and social isolation can increase the likelihood of mental disorders such as depression, anxiety, substance use, and cognitive decline [7].

Loneliness undermines people's ability to self-regulate and represents the pain of feeling alone [8].

Home office routine requires regular evaluation of the individual´s ability to withstand repetitive work at home and the lack of contact with coworkers to talk in person. Individuals also tend to feel disconnected from the organization or group to which they belong. It is possible to lose track of time due to not sharing time with others, all aggravated by excessive use of digital technologies, reinforcing the solitary nature of home office work, which can lead to unhealthy behavior changes [9].

The current study aimed to evaluate the results and impacts according to the sample´s demographic characteristics, seeking to identify specific human behaviors in home office work conditions during the COVID-19 pandemic. The hypothesis is that work conditions in home office format can alter humans´ perception of their work. The study used a statistically validated scale to test this hypothesis.

## 2 MATERIALS AND METHODS

Considering the novelty and emergence of the study topic, the Home Office Work Scale (HOWS) was applied, validated and published in *Mental Health and Addiction Research* in 2021 (doi:10.15761/MHAR.1000201). Our sample consisted of 1,056 valid questionnaires [9].

The keywords were: home office; coronavirus; COVID-19; pandemic; human behavior; social distancing; social isolation. The same keywords used in the search in the questionnaires were used in the published manuscript that validated the HOWS scale [9].

### 2.1 Data Collection

The Home Office Work Scale (HOWS) was applied to situations with people in social distancing, working at home.

 The survey initially included a representative sample of 1,083 volunteers of both sexes, 18 to 70 years of age, divided into seven age groups. We excluded 27 questionnaires due to completion errors, resulting in a final sample of 1,056 participants. Data were collected online through the authors´ digital channels using a structured computational tool (Google Forms). The questionnaire remained available for 45 days (06.15.2020 to 07.30.2020), which determined the sample size. The target audience consisted of persons working exclusively in a home office format during the COVID-19 pandemic.

 The HOWS scale (Annex **1**) consisted of 10 questions with detailed instructions for volunteers, with the following options: no (0); yes, a little (1), or yes, a lot (2) to verify respondents´ perception of their home office experience.

 To reduce possibilities of research bias, detailed instructions for volunteers were described at the beginning of the scale (Annex 1).

 There is no specific law or guidelines that regulate this kind of research method in Brazil, as with face-to-face collections.

### 2.2 Inclusion Criteria

Respondents of both sexes who were working at home, belonging to different age groups, established in the survey, and using digital access without face-to-face contact with coworkers, superiors, or clients.

### 2.3 Exclusion Criteria

Volunteers working either in person at their regular workplace or in a hybrid format (some days at home and some days in person at their regular workplace).

## 3 RESULTS

### 3.1 Data

There were errors in the completion of 27 questionnaires that required excluding those participants, decreasing the sample from 1,083 to 1,056 valid questionnaires.

### 3.2 Descriptive Analysis

The statistical analysis consisted of a demographic analysis of the prevalence of participation between women and men and age groups for testing the hypothesis.

In Table **1**, the sample's descriptive statistics showed greater participation by women (58% females and 42% males). The age groups from 18 to 25 and from 66 to 70 years showed the lowest participation rates, while the other age groups were balanced [10, 11].

### 3.3 Test of Hypothesis

Student's t-test was applied to the mean difference between males and females, aimed at verifying whether men and women behaved differently at work in a home office format during the pandemic, which did not occur (Table **2**).

Since the p-value exceeded 0.05, statistically, we do not reject the hypothesis that the mean of the groups is the same. That is, independently of sex, there was no evidence for further investigating whether men and women behaved differently, although the number of women was higher.

*Cohen's “d”* is equal to 0.11, indicating a small effect size in the *t-test*, confirming that the difference in results between men and women is almost nil.

### 3.4 Frequency of Responses

In Table **3**, the percentages and absolute values ​​for each question, distributed across the three options (no, yes, a little, and yes, a lot), allowed an assessment of volunteers´ views concerning working in a home office format during social distancing.

## 4 DISCUSSION

### 4.1 ParticiPation According to Gender and Age

Descriptive statistics such as summary measures (Table **1**) showed higher participation by women (58%) than men (42%), corroborating other research findings that show higher female presence in online surveys. Despite this higher percentage, the *t-test* showed that the mean values between men and women were quite close. Cohen´s *d* was 0.11, indicating a small effect size in the t test, *i.e*., the difference between the results for men and women was practically nil.

Another survey (n=589) analyzing patterns in smartphone use by men and women found an increase in the level of use by women when compared to men. Women tended to use more social networks and access more messaging features such as e-mails and apps. Gender was thus considered statistically significant in terms of smartphone use [12].

According to another study, variables such as gender, family, activity, use of smartphones, and frequency and direction of use were correlated with problems arising from excessive use of these technologies [13].

A survey in which 70.9% of the participants were women found that the relationship between the pursuit of goals and the fear of being without their online devices determined the impacts according to gender [14].

### 4.2 Test of Hypothesis

The extreme age groups, 18 to 25 and 66 to 70 years, showed the lowest participation, respectively 3.4% and 6.4%, with low significance, to be investigated in future research, since it was not among the current study´s objectives.

The largest participation was found in the three central age groups (34 to 41, 42 to 49, and 50 to 57 years), which totaled 61.9% of the respondents, especially the 42-49-year group with more than 22% of participants.

The demographic data showed consistent frequency and offered satisfactory variability, thereby minimizing the tendency for one age group to dominate (which might have created a statistical bias). Future research using this scale should allow observing behaviors with a predominance of age groups, thus making another contribution from the scale´s use.

### 4.3 Frequency of Responses

According to question 1, only 19% of respondents reported any experience with home office work before social distancing during the COVID-19 pandemic. Even so, only 16% had to adapt their work systems (question 2). Only 12% reported that they had to adapt their furniture at home for continuing online work and to resemble their regular workplace.

Future architectural projects should include spaces suitable for the home office and not merely a room with a bookcase and a table and sofa for watching television. Such spaces will have to offer a physical infrastructure with furniture and layout that allow the work to be performed in conditions closer to those available in the individual´s regular workplace, *e.g*., in their offices in company buildings [15].

Digital ergonomics must be considered in these spaces, due to the risk of physical injuries related to improper posture and inappropriate furniture when using digital devices at home [16].

This may explain why fewer than half (49%) of the respondents reported that they felt comfortable with their home office work conditions (question 4).

One-fourth of respondents (25%) reported physical and emotional changes (question 5) due to work in the home office format. However, 51% felt some changes due to the home office format during social distancing.

Pandemics and epidemics can affect people's physical and emotional health and disrupt society, usually resulting in a high level of psychological distress and psychosocial maladjustment [1].

From a health perspective, the home office can create various problems, including a sedentary lifestyle, overweight, and failure to take snack breaks or simply having a cup of coffee instead. In the regular physical and in-person workplace, such breaks are healthy, and they energize workers. The same is not necessarily true at home unless the worker is highly disciplined.

Only 31% of our sample reported that their work performance improved in the home office (question 6). More than one-third of the respondents (37%) stated that they had not seen any improvement in their work performance.

Due to rules by some companies that require employees´ constant availability, wherever they are, workers´ well-being becomes fundamentally relevant to organizational results. It is necessary to take proper care of the employees' health [17].

Concerning the impacts of home office work on domestic activities, 45% of the respondents reported some impact (question 7), although 50% reported having maintained their routine within the normal range (question 8).

Impacts on human behavior are relevant when changes occur in individuals´ routine, requiring changes in habits and relations, especially when isolation is imposed with the intensification of online practices [9].

Eating disorders comprise a group of conditions characterized by persistent disturbances in eating or in related behaviors, resulting in altered consumption or absorption of food, impairing physical health and psychosocial functioning.

Home office work tends to situate individuals too close to the refrigerator and pantry. Staying at home requires considerable determination to adhere to mealtimes and to choose what to eat and in what quantities. Surprisingly, people may consume more ice cream and other high-fat foods when sitting at home feeling totally alone in the world [10].

Notwithstanding the current impacts, 39% of respondents reported in question 9 that they may continue home office work after the pandemic´s social distancing ends. In the same question, 33% reported that they believe they will continue to work part of the time in home office format [18].

Finally, 45% answered (question 10) that home office work is a solution for all professions.

Despite numerous benefits to users of information technologies, the adverse effects of the indiscriminate use of technological devices in work environments is a topic that merits further study [18].

Information and communication technologies can change the ways people relate to others, with both positive and negative effects. It all depends on how people use or abuse them [5].

A home office may increase the number of work hours due to the lack of control in the administration of time at home, which does not include the breaks normally practiced in the regular workplace.

### 4.4 Limitations

It is important to highlight the topic´s original nature, the need to develop a scale due to the lack of other equivalent scales, based on the immediate experience created by an unforeseen pandemic and the lack of knowledge on this type of evaluation.

Home office work with the freedom to come and go is nothing new. However, social distancing with increased use of digital technologies and with no option to leave home imposes different conditions for home workers, which can be a limitation with respect to performance, if compared to performance in the in-person workplace.

The definition of what home office work represents for people in social distancing may differ in relation to more flexible work that allows part of the work to be performed in the regular workplace.

## CONCLUSION

More women than men participated in this survey on the use of digital technologies, although the results did not differ greatly between women and men. The extreme age groups (18 to 25 and 66 to 70 years) showed low participation rates and low significance, which merits further study. Among the response rates, many respondents reported continuing to work at home even with the changes they needed to make in their routines.

The unprecedented scale reported here may serve future scientific studies under the conditions described in this article, which translates as external validity.

The growing interest in this topic and its possible effects on organizational culture, human behavior, and workplace operations, in general, justifies this endeavor. Further research should reinforce the scale´s validity and may allow the comparison between results produced by individuals under the conditions reported in this study [9].

## AUTHORS´ CONTRIBUTIONS

L L Gonçalves reviewed the literature, applied the scales, and wrote the article.

A E Nardi co-oriented and reviewed the article.

H K Santos performed the statistical analysis and wrote the article.

D Rodrigues performed the statistical analysis and wrote the article.

A L S King oriented the study and reviewed and wrote the article.

## Figures and Tables

**Table 1:** Descriptive statistics participation of women and men and age groups (in years).

**Men**				**Women**			
441 **(42%)**				615 **(58%)**			
**Age Groups**							
**18 to 25**	**26 to 33**	**34 to 41**	**42 to 49**		**50 to 57**	**58 to 65**	**66 to 70**
**36** **(3.4%)**	150 (14.2%)	209 (19.8%)	241 (22.8%)		204 (19.3%)	148 (14.0%)	**68** **(6.4%)**

**Table 2:** Test between men and women.

**Mean (Standard Deviation)**		**T**	**p-value**
Men	Women		
9.75 (3.127)	10.252 (3.35)	-1.03	0.057

**Table 3:** Frequency of responses (absolute and relative) 1,056 volunteers.

				**Focus of the Question**
**Question**	**0**	**1**	**2**	
**Q1**	431 (41%)	424 (40%)	201 (19%)	Have you worked in HO before social distancing?
**Q2**	370 (35%)	520 (49%)	166 (16%)	Have you adapted to the HO work system?
**Q3**	454 (43%)	475 (45%)	127 (12%)	Did you need to adapt the furniture?
**Q4**	143 (14%)	392 (37%)	521 (49%)	Is HO comfortable?
**Q5**	252 (24%)	538 (51%)	266 (25%)	Have you experienced physical/emotional changes?
**Q6**	386 (37%)	344 (33%)	326 (31%)	Were there work improvements in HO?
**Q7**	344 (33%)	470 (45%)	242 (23%)	Has HO impacted your domestic activities?
**Q8**	134 (13%)	399 (38%)	523 (50%)	Have you maintained a routine in HO?
**Q9**	299 (28%)	349 (33%)	408 (39%)	Will you continue with HO after social distancing?
**Q10**	226 (21%)	471 (45%)	359 (34%)	Will HO continue for everyone?

HO = Home Office

## Data Availability

The data supporting the findings of the article is available within the article.

## Assessment of Individuals´ Behavior in Home Office (HO) Work during Social Distancing in the COVID-19 Pandemic

This questionnaire aims to assess the impact on individuals who began to work in the Home Office (HO) format during social distancing and to identify their perspectives regarding the continuation of this type of work after the novel coronavirus pandemic (COVID-19). The survey will be available for access from June 15, 2020, to July 30, 2020.

HO activities are defined here as work that would normally be performed in a company or workplace or as a self-employed person or in informal work on the streets and began to be performed at home during the period of social distancing in the COVID-19 pandemic.

We invite volunteers 18 to 70 years of age to participate in this online survey on work in the Home Office (HO) during social distancing, regardless of professional profile. Not included are individuals performing work in person or in a hybrid format (part of the time at their regular workplace and part of the time at home). There are 10 questions that will require about 5 minutes to answer.

We guarantee the anonymity of respondents, who will be contributing to the Delete Laboratory - Digital Detox and Conscious Use of Technologies of the Institute of Psychiatry (IPUB) of the Federal University of Rio de Janeiro (UFRJ) in developing appropriate strategies for understanding human behavior in the new times of the digital world and well-being in times of crisis.

## Identification - Gender

() Female

() Male

## Identification Age group (years)

() 66 to 70

Options

Note: **HO** =r **Home Office**

Choose only one option:

No = 0

Yes, a little = 1

Yes, a lot = 2

After you finish, add up your points. According to your answers, you will see if your prospects for continuing to work in the Home Office format after the end of social distancing are low, medium, or high.

You may edit and print your answers if you want.

## Questions:

1. Had you ever worked, even occasionally, in a Home Office format before social distancing?

2. Did you have to make adaptations to your work system to make it accessible in HO during social distancing?

3. Have you adapted your furniture to accommodate Digital Ergonomics (posture and furniture) suitable to work in HO (physical space, table, Internet, ambient lighting, support for monitors, and seating suited to your physical conditions)?

4. Do you feel comfortable, in general terms, at work in HO?

5. Have you noticed any physical and or emotional changes at work in HO?

6. Have you noticed improvements in your productivity working in HO?

7. Have your domestic activities been impacted by working in HO?

8. Were you able to maintain a home office work routine and adhere to your schedule during the social distancing period?

9. Do you consider it possible to maintain your professional activity in home office after the pandemic?

10. Do you consider HO work a permanent solution for most professional activities?

## Results

The sum of points from your answers indicates the range of your results. Higher scores indicate better adaptation to the Home Office, which may be a viable alternative for work after the end of social distancing.

**0 to 6 points: LOW PROSPECTS** - The experience in Home Office has not seemed comfortable for you and may not be a positive alternative for your professional life. The most suitable solution for your profile would be to return to in-person work after the end of social distancing.

**7 to 13 points: MEDIUM PROSPECTS** - Your experience with Home Office demonstrated an intermediate position for the adoption of this way of working, requiring an evaluation of the aspects less adapted to this approach and proposals for improvements in case you intend to adopt this system after the end of social distancing.

**14 to 20 points: HIGH PROSPECTS** - Your adaptation to the Home Office system was relatively easy, and this type of work may become permanent in your professional life after the end of social distancing.

The Delete Laboratory/ IPUB / UFRJ team thanks you for your participation.

For more information and guidance on the excessive use of technologies in everyday life and for digital addiction treatments, feel free to contact us at the following addresses:

E-mails: grupodelete@gmail.com or annaluciaking@gmail.com

Facebook: Delete - Digital Detox and Conscious Use of Technologies

For guidance on organizational, physical, and psychological implications related to work in the Home Office, kindly contact the Delete team at the following e-mails: luciolage@msn.com (organizational) and annaluciaking@gmail.com (psychological).
